# A brainstem circuit for nausea suppression

**DOI:** 10.1016/j.celrep.2022.110953

**Published:** 2022-06-14

**Authors:** Chuchu Zhang, Lindsay K. Vincelette, Frank Reimann, Stephen D. Liberles

**Affiliations:** 1 Howard Hughes Medical Institute, Department of Cell Biology, Harvard Medical School, Boston, MA 02115, USA; 2 Wellcome Trust - MRC Institute of Metabolic Science, Metabolic Research Laboratories, Addenbrooke’s Hospital, Hills Road, Cambridge CB2 0QQ, UK; 3 Lead contact

## Abstract

Nausea is a discomforting sensation of gut malaise that remains a major clinical challenge. Several visceral poisons induce nausea through the area postrema, a sensory circumventricular organ that detects blood-borne factors. Here, we use genetic approaches based on an area postrema cell atlas to reveal inhibitory neurons that counteract nausea-associated poison responses. The gut hormone glucose insulinotropic peptide (GIP) activates area postrema inhibitory neurons that project locally and elicit inhibitory currents in nausea-promoting excitatory neurons through γ-aminobutyric acid (GABA) receptors. Moreover, GIP blocks behavioral responses to poisons in wild-type mice, with protection eliminated by targeted area postrema neuron ablation. These findings provide insights into the basic organization of nausea-associated brainstem circuits and reveal that area postrema inhibitory neurons are an effective pharmacological target for nausea intervention.

## In brief

Nausea is an unpleasant sensation of visceral malaise that remains poorly understood at a molecular and cellular level. Zhang et al. uncover brainstem inhibitory neurons that suppress nausea-related behaviors through selective blockade of nausea-promoting, GDF15 responsive-excitatory neurons. Nausea-suppressing neurons can be pharmacologically engaged by the gut hormone glucose insulinotropic peptide (GIP).

## Graphical abstract

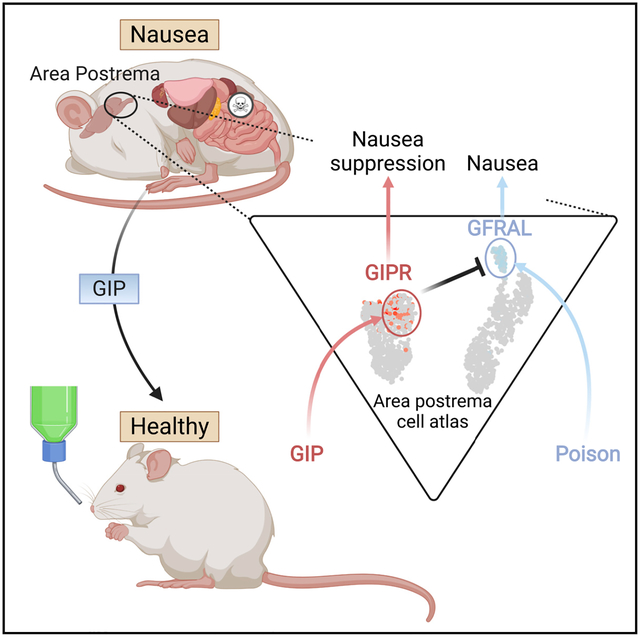

## INTRODUCTION

Nausea is one of the most encountered symptoms in healthcare, with current anti-nausea medications displaying variable clinical success (Hesketh, 2008). New strategies for nausea intervention are needed and may be enabled by a mechanistic understanding of how the sensation of nausea arises. Classical studies involving brain lesion and stimulation revealed a tiny brainstem structure termed the area postrema that mediates nausea responses to several visceral threats (Borison, 1989). The area postrema is a sensory circumventricular organ with a privileged anatomical location containing a reduced blood-brain barrier, which allows resident neurons to sample hormones and other chemicals in the circulatory system (Borison, 1989).

Single-cell cDNA sequencing approaches recently provided a cell atlas of the area postrema, revealing four excitatory and three inhibitory neuron types (Zhang et al., 2021). One excitatory neuron type expresses multiple receptors for nausea-inducing stimuli, including the growth/differentiation factor 15 (GDF15) receptor (GFRAL), the glucagon-like peptide 1 (GLP1) receptor (GLP1R), and the calcium-sensing receptor (CaSR). GFRAL, GLP1R, and CaSR agonists cause nausea and/or vomiting in large animals and in mice, which are incapable of vomiting, evoke a characteristic behavioral response termed conditioned flavor avoidance in which paired administration of a poison and a novel flavor causes future avoidance of that flavor (Andrews, 1992; Patel et al., 2019; Zhang et al., 2021). Chemogenetic activation of area postrema GFRAL neurons promotes flavor avoidance, while their ablation eliminates avoidance imposed by several visceral poisons (Sabatini et al., 2021; Zhang et al., 2021). Thus, area postrema GFRAL neurons are a key node in nausea circuits, and inhibitory pathways that lower their activity may reduce symptoms of gut malaise.

The functions of area postrema inhibitory neurons are unclear. All three area postrema inhibitory neuron types (and none of the excitatory neurons) are marked in *Gad2-ires-Cre* mice, and genetic tracing previously revealed their projections to be local and largely confined to the area postrema, with minor projections observed in the adjacent nucleus of the solitary tract (NTS) and not in other brain regions that receive excitatory area postrema inputs (Zhang et al., 2021). Based on this observation, we hypothesized that at least some inhibitory neuron types may suppress the activity and function of area postrema excitatory neurons, including those involved in nausea.

## RESULTS

### Area postrema inhibitory neurons suppress local excitatory neurons and poison responses

**Figure 1.**
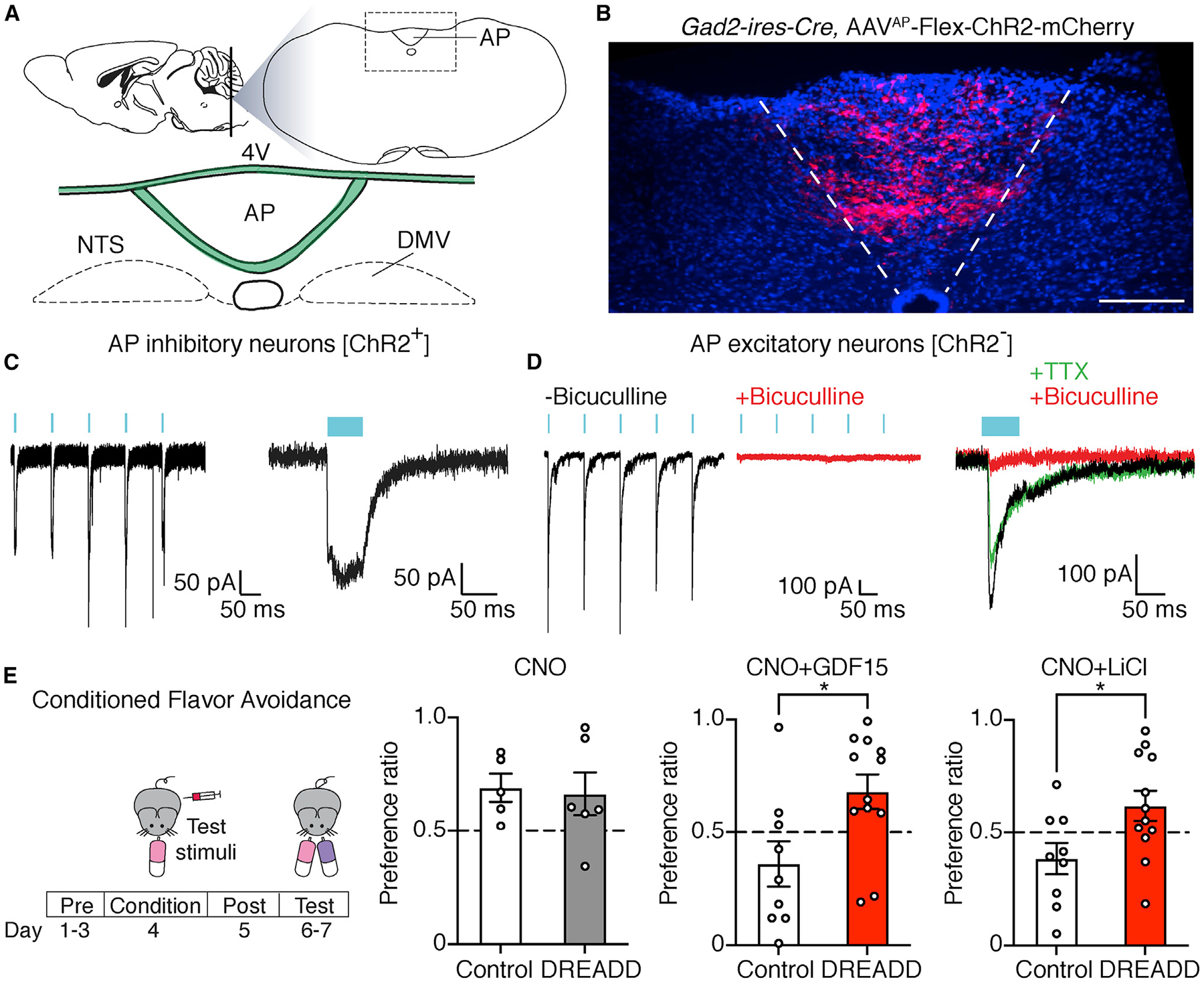
Area postrema inhibitory neurons suppress local excitatory neurons and nausea-associated behavior (A) Cartoon indicating area postrema (AP) location in the brainstem. 4V, fourth ventricle; DMV, dorsal motor nucleus of the vagus; NTS, nucleus of the solitary tract. (B) Native mCherry fluorescence in coronal area postrema cryosections of *Gad2-ires-Cre* mice with area-postrema-localized injection of AAV-Flex-ChR2-mCherry. Scale bar: 100 μm. (C) Whole-cell, voltage-clamp recordings of responses to light (blue bar) in mCherry-expressing area postrema neurons in brainstem slices from *Gad2-ires-Cre*; AAV-Flex-ChR2-mCherry mice. Responses were observed in 5/5 mCherry-positive neurons from 4 mice. (D) Light-induced postsynaptic currents were measured in GFP-negative area postrema neurons from *Gad2-ires-Cre, Rosa26-lsl-L10GFP* mice previously injected in the area postrema with AAV-Flex-ChR2-mCherry. Whole-cell, voltage-clamp recordings of photostimulation-induced IPSCs were made at −60 mV using a high-chloride intracellular solution to reveal a chloride conductance. Some recordings were made in the presence of bicuculline (10 μm, red) or tetrodotoxin (TTX; 100 nM, green). Responses observed in 16/18 neurons, 5 mice. (E) *Gad2-ires-Cre* mice were injected in the area postrema with (DREADD) or without (control) AAV-Flex-GsDREADD-mCherry. CNO was administered on the flavor-conditioning day prior to LiCl (right), GDF15 (middle), or saline injection (left), and the effect on subsequent flavor avoidance was measured. n (left to right) = 5, 6, 9, 12, 9, and 12; mean ± SEM; circles: individual mice; Mann-Whitney test, *p < 0.05.

We used channelrhodopsin (ChR2)-assisted circuit mapping (CRACM) to investigate the connectivity patterns of area postrema inhibitory neurons. *Gad2-ires-Cre, Rosa26-lsl-L10GFP* mice were injected in the area postrema with an AAV containing a Cre-dependent ChR2-mCherry allele (AAV-Flex-ChR2-mCherry); post hoc histological analysis confirmed that viral infection was largely restricted to the area postrema (Figures 1A and 1B). Whole-cell recordings in area postrema tissue slices revealed robust light-gated currents in mCherry-positive inhibitory neurons (Figure 1C). Post-synaptic responses were then measured in area postrema excitatory neurons, which were identified by neuronal morphology and a lack of GFP fluorescence in *Gad2-ires-Cre, Rosa26-lsl-L10GFP* mice. Optogenetic activation of area postrema inhibitory neurons produced large outward chloride currents (using high-chloride intracellular solution) in area postrema excitatory neurons (resting membrane potential: −49.3 ± 1.1 mV; Figure S1A) that were abolished by application of the GABA_A_ receptor antagonist bicuculline (Figure 1D). Light-evoked inhibitory post-synaptic currents (IPSCs) in most excitatory neurons (89%, 16/18) displayed onset kinetics and insensitivity to tetrodotoxin (Figures 1D and S1B), consistent with a direct monosynaptic connection from inhibitory neurons. Post-synaptic responses were also observed with reduced frequency in 27% (6/22) of area postrema inhibitory neurons (mCherry-negative, GFP-positive neurons) and 38% (8/21) of excitatory neurons in adjacent NTS regions (Figures S1C and S1D). Together, these findings indicate that most area postrema excitatory neurons receive local inhibitory input, with area postrema inhibitory neurons also forming functional connections with some NTS excitatory neurons and other area postrema inhibitory neurons.

We activated area postrema inhibitory neurons using chemogenetic approaches and observed the consequences on mouse behavior. We injected *Gad2-ires-Cre* mice in the area postrema with or without adeno-associated viruses (AAVs) containing Cre-dependent genes encoding designer Gα_s_-coupled receptors (AAV-Flex-GsDREADD-mCherry) activated by the synthetic agonist clozapine-*N*-oxide (CNO) (Roth, 2016); Gα_s_-coupled receptors were used based on commonly expressed area postrema receptors (see below). Chemogenetic activation of area postrema inhibitory neurons was validated by measuring CNO-induced Fos expression in mCherry-labeled neurons (Figure S2A). Proper AAV targeting of the area postrema was confirmed in every animal post hoc by blinded histological analysis of mCherry expression; we sometimes observed targeting of a few nearby NTS neurons (Figure S2B), and animals lacking labeled area postrema neurons were excluded from analysis (see STAR Methods for more information).

We used an established behavioral paradigm to measure conditioned flavor avoidance (Patel et al., 2019; Zhang et al., 2021). Briefly, on a conditioning day, water-restricted mice were given access to a novel flavored saccharin solution (either cherry or grape flavored) and then immediately injected with saline (control), poisons, and/or CNO. Subsequently, on a testing day, behavioral preference for cherry- or grape-flavored solution was measured using a two-choice assay and expressed as a preference index where the time drinking the conditioned flavor was divided by total time drinking. In the absence of malaise induction, mice displayed a modest preference for the experienced flavor, with similar results observed for cherry and grape (Zhang et al., 2021). In contrast, robust behavioral avoidance of the experienced flavor was evoked by various poisons (Zhang et al., 2021), as well as the GFRAL agonist GDF15, as reported previously (Patel et al., 2019). CNO-induced activation of area postrema inhibitory neurons did not evoke flavor aversion or attraction in this paradigm in the absence of poison induction (Figure 1E). Strikingly, however, when chemogenetic activation of area postrema inhibitory neurons was paired with GDF15 or lithium chloride injection, imposed aversion was lost (Figure 1E). CNO silenced GDF15 and lithium chloride responses in mice with DREADD expression in inhibitory neurons but had no effect in control mice lacking DREADD expression, either in the presence or absence of poisons. Together with anatomical and connectivity-mapping studies, these findings indicate that at least some area postrema inhibitory neurons project to and inhibit the activity and function of nausea-promoting excitatory neurons.

### GIP inhibits GFRAL neurons through a monosynaptic connection

We focused on one subpopulation of area postrema inhibitory neurons that express the receptor (GIPR) for glucose insulinotropic peptide (GIP). GIP is a gut-derived hormone and incretin released upon nutrient intake that rapidly promotes insulin release (Baggio and Drucker, 2007). Small molecules that activate the receptor for another incretin, GLP1, are clinical mainstays for diabetes treatment but induce nausea as an adverse side effect through area postrema excitatory neurons (Drucker and Nauck, 2006; Zhang et al., 2021). Recent studies involving paired administration of both incretins—GIP and GLP1— observed that GIP suppressed some adverse behavioral responses to GLP1 (Borner et al., 2021); furthermore, GIPR agonists reduced morphine- and cancer-drug-induced vomiting in ferrets (Asami et al., 2018). The neuronal basis for GIP responses remains unclear, with several gut-brain communication routes possible.

**Figure 2.**
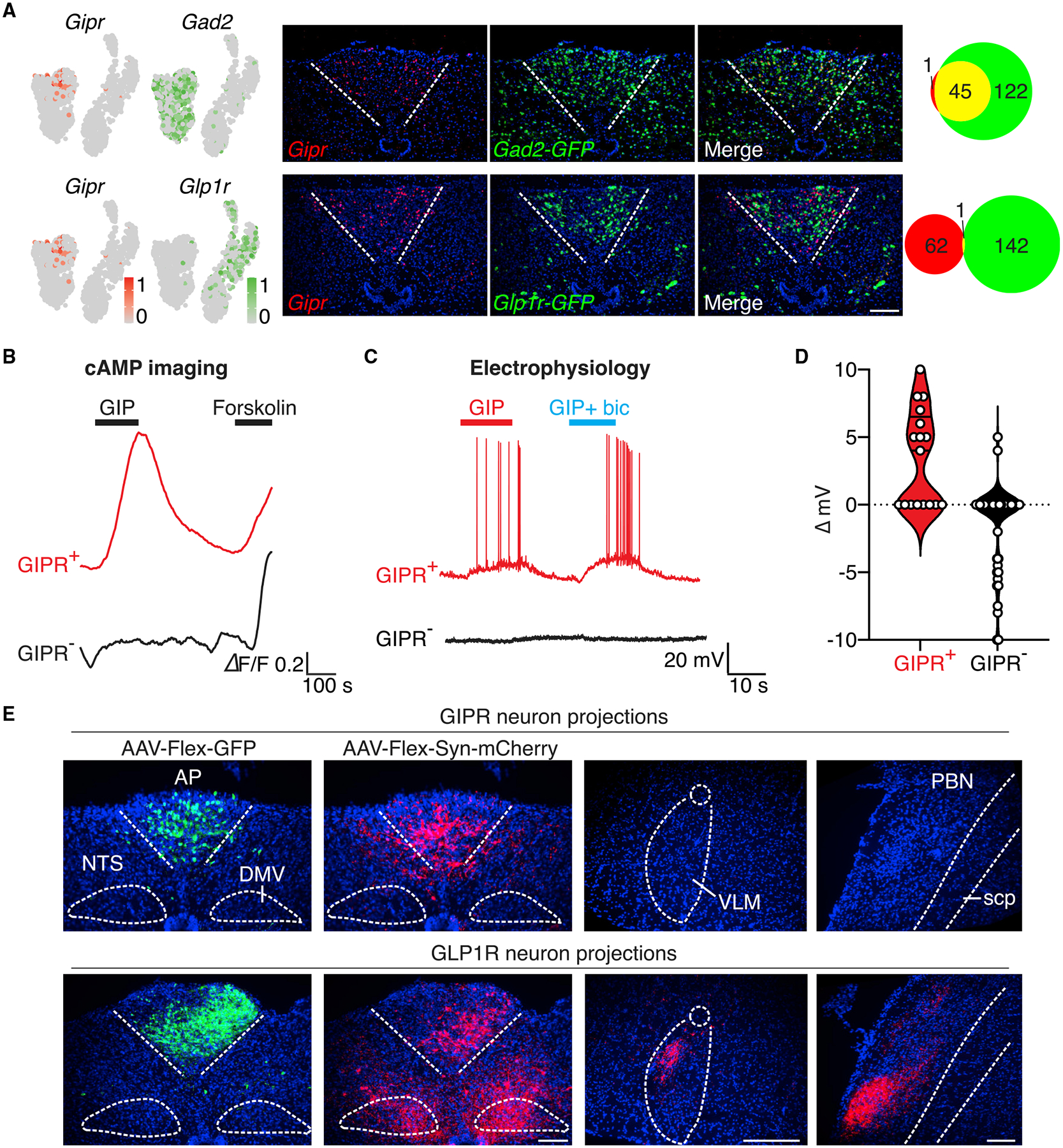
GIP activates area postrema inhibitory neurons with local projections (A) Expression of *Gipr* was compared with *Gad2* (top) and *Glp1r* (bottom). Uniform manifold approximation and projection (UMAP) plots (left) are derived from published single-cell transcriptome data (Zhang et al., 2021). Two-color expression analysis (middle) was performed in coronal area postrema cryosections of *Gad2-ires-Cre*; *Rosa26-lsl-L10GFP* (top) and *Glp1r-ires-Cre*; *Rosa26-lsl-L10GFP* (bottom), and involved fluorescent RNA *in situ* hybridization for *Gipr* (red) and native GFP fluorescence (green). Scale bar: 100 μm. Co-labeled (yellow) or individually labeled (red, green) area postrema neurons were counted (right) from images in middle. (B) cAMP transients evoked by GIP (100 nM) and forskolin (25 mM) were measured in tdTomato-positive (GIPR^+^, red) and tdTomato-negative (GIPR^−^, black) area postrema neurons cultured from *Gipr-Cre; Rosa26-lsl-tdTomato* mice. (C) Whole-cell, current-clamp responses of tdTomato-positive (GIPR^+^, red) and tdTomato-negative (GIPR^−^, black) area postrema neurons in brainstem slices from *Gipr-Cre; Rosa26-lsl-tdTomato* mice to GIP (100 nM) with ionotropic glutamate receptor (iGluR) antagonists CNQX (10 μM) and D-AP5 (50 μM) or GABA_A_ receptor antagonist bicuculline (bic, 10 μM). (D) GIP-evoked changes in membrane potential in tdTomato-positive (left, GIPR^+^, red) or tdTomato-negative (right, GIPR^−^, black) neurons from recordings in (C). n= 17 (left) and 74 (right); circles: individual data points. (E) AAV-Flex-GFP and AAV-Flex-Syn-mCherry were injected into the area postrema of *Gipr-Cre* (top) and *Glp1r-ires-Cre* (bottom) mice. GFP-positive neurons and mCherry-positive synaptic terminals were visualized by immunohistochemistry. A GFP fluorescence intensity was used, which enables visualization of neuronal cell bodies but not axons. VLM, ventrolateral medulla; PBN, parabrachial nucleus; scp, superior cerebellar peduncle. Scale bar: 100 μm.

We previously revealed by single-cell cDNA sequencing that *Gipr* is expressed in a subset of area postrema inhibitory neurons (neuron cluster 6; Figure 2A) (Zhang et al., 2021). Since we observed here that area postrema inhibitory neurons suppress poison responses, we hypothesized that cluster 6 neurons may directly mediate or contribute to the anti-nausea effects of GIP and that targeting these neurons could represent a general strategy for nausea intervention. Moreover, GIPR expression provides a selective molecular handle for both genetic and pharmacological control of cluster 6 neurons. We obtained *Gipr-Cre* mice (Adriaenssens et al., 2019) and validated efficient targeting of cluster 6 area postrema neurons by two-color expression analysis involving dual visualization of a Cre-driven fluorescent reporter and *Gipr* mRNA by *in situ* hybridization (Figure S3A); as is common with Cre tools, we noted some reporter-positive, *Gipr*-negative cells, more so in the NTS than the area postrema, which may have resulted from *Gipr* expression that is transient or too low for detection by RNA *in situ* hybridization.

We asked whether GIP evoked responses in area postrema cluster 6 neurons marked in *Gipr-Cre, R26-lsl-tdTomato* mice; as a note, we used [D-Ala^2^]-GIP as a stable GIP analog in all experiments. GIPR is a Gα_s_-coupled receptor, and we observed GIP-evoked cAMP transients in dissociated GIPR neurons (71.4%, 15/21 cells) using the genetically encoded fluorescent cAMP sensor cADDis (Figure 2B) (Tewson et al., 2016). Increasing cAMP levels depolarizes some, but not all, neurons, so we asked whether GIP also evokes electrical responses in cluster 6 neurons. Whole-cell, patch-clamp analysis in area postrema slices revealed robust GIP-evoked depolarization and action potentials in tdTomato-positive neurons (9/17 cells) from *Gipr-Cre, R26-lsl-tdTomato* mice (Figures 2C and 2D). For comparison, GIP rarely depolarized (2/74 cells) or evoked cAMP transients (3/29 cells) in other area postrema neurons lacking tdTomato expression; instead, hyperpolarization of some tdTomato-negative neurons was observed (23%, 17/74 neurons). Intraperitoneal (i.p.) injection of GIP also induced Fos expression in area postrema neurons, with effects persisting after vagotomy, suggesting that vagal inputs were not required for area postrema responses to GIP (Figure S3B). Taken together, GIP evokes characteristic and direct responses in GIPR-expressing area postrema inhibitory neurons that include increased cAMP levels, depolarization, and cell firing.

We used genetic approaches in *Gipr-Cre* mice to map the anatomy of area postrema cluster 6 neurons and, for comparison, used *Glp1r-ires-Cre* mice to map area postrema excitatory neuron clusters 2–4 (Figure 2E) (Williams et al., 2016; Zhang et al., 2021). We injected AAV-Flex-GFP and AAV-Flex-Synaptophysin-mCherry into the area postrema to label neuronal cell bodies and synaptic terminals, respectively. As we observed previously, area postrema excitatory neurons project to multiple brain regions including the NTS, parabrachial nucleus (PBN), and autonomic motor nuclei (Zhang et al., 2021). In contrast, we observed that area postrema GIPR neurons, like inhibitory neurons in bulk, displayed dense arborizations within the area postrema and minor projections to proximal NTS, but GIPR neuron projections were not observed in the PBN or autonomic motor nuclei. These findings raised the possibility that GIPR neurons form inhibitory contacts with some or all area postrema excitatory neurons.

**Figure 3.**
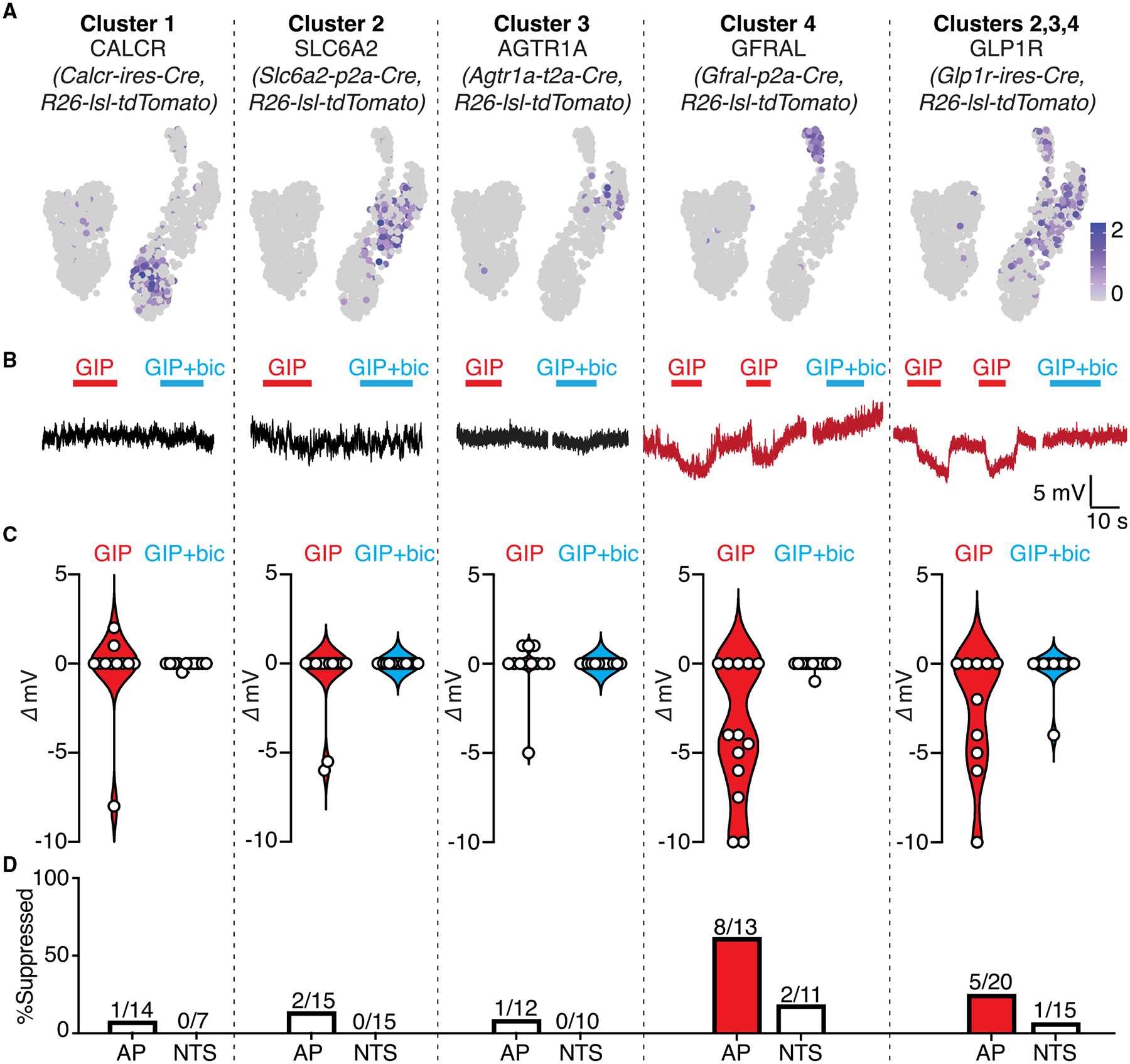
GIPR neurons selectively inhibit area postrema GFRAL neurons (A) UMAP plots derived from published single-cell transcriptome data (Zhang et al., 2021) indicate expression of marker genes in different area postrema excitatory neurons. (B) Whole-cell, current-clamp responses to GIP (100 nM) of tdTomato-positive area postrema neurons in brainstem slices from either (1) *Calcr-ires-Cre*; *Rosa26-lsl-tdTomato*, (2) *Slc6a2-p2a-Cre; Rosa26-lsl-tdTomato*, (3) *Agtr1a-t2a-Cre; Rosa26-lsl-tdTomato*, (4) *Gfral-p2a-Cre; Rosa26-lsl-tdTomato*, or (5) *Glp1r-ires-Cre; Rosa26-lsl-tdTomato* mice. Recordings were done with either iGluR antagonists (red) CNQX (10 μM) and D-AP5 (50 μM) or GABA_A_ receptor antagonist bic (10 μM, blue). (C) Maximal GIP-evoked membrane potential change with (blue) or without (red) bic from individual neurons (circles) from (B). (D) Frequency of neurons showing GIP-induced hyperpolarization from area postrema and NTS in mice from (B).

We asked whether area postrema cluster 6 neurons evoked inhibitory postsynaptic currents in particular classes of area postrema excitatory neurons. Various excitatory neuron types were marked in *Calcr-ires-Cre; Rosa26-lsl-tdTomato* (cluster 1), *Slc6a2-p2a-Cre; Rosa26-lsl-tdTomato* (cluster 2), *Agtr1a-t2a-Cre; Rosa26-lsl-tdTomato* (cluster 3), *Gfral-p2a-Cre; Rosa26-lsl-tdTomato* (cluster 4), and *Glp1r-ires-Cre; Rosa26-lsl-tdTomato* (clusters 2–4) mice. We obtained area postrema tissue slices from mice of each genotype and examined GIP-evoked responses by whole-cell, patch-clamp electrophysiology in the presence of ionotropic glutamate receptor antagonists (D-AP5, CNQX) to block excitatory transmission (Figure 3). GIP induced acute hyperpolarization (−6.4 ± 0.9 mV) of GFRAL-expressing area postrema neurons (8/13 cells) but failed to hyperpolarize most other excitatory neurons (SLC6A2: 2/15 cells; AGTR1A: 1/12 cells, CALCR: 1/14 cells). GIP hyperpolarized 20% (4/20) of GLP1R neurons, consistent with GLP1R expression in multiple neuron types with differing GIP sensitivity. The GABA_A_ receptor antagonist bicuculline did not impact GIP-evoked depolarization of GIPR neurons but did eliminate GIP-evoked hyperpolarization of GFRAL neurons. Taken together, these findings indicate that GIP directly stimulates area postrema cluster 6 neurons, which in turn form local and selective GABAergic inhibitory connections with area postrema GFRAL neurons (cluster 4).

### GIP suppresses nausea behaviors through area postrema inhibitory circuits

**Figure 4.**
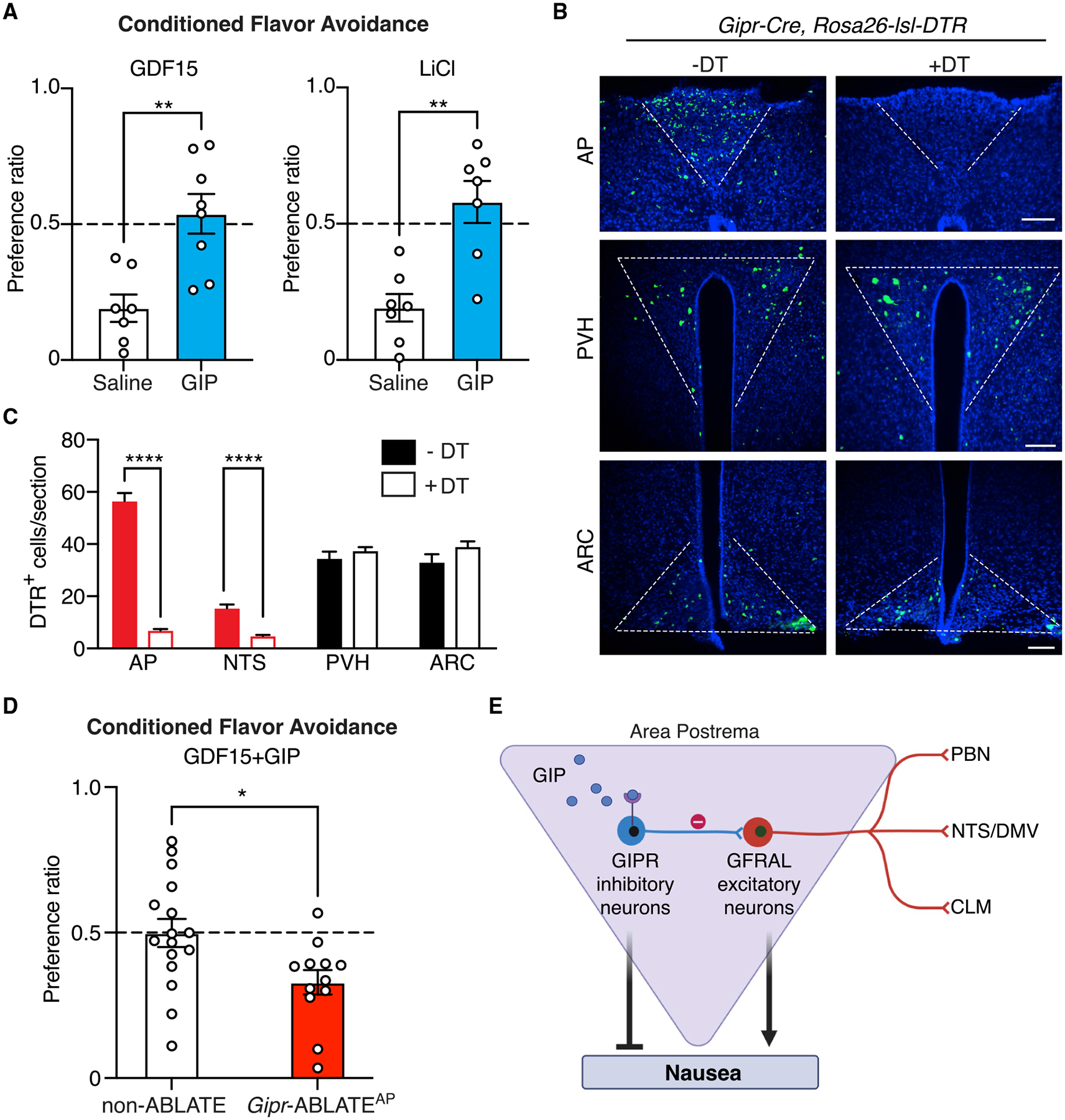
GIP suppresses nausea-associated behavior through area postrema inhibitory circuits (A) Flavor avoidance conditioned by GDF15 (left) or LiCl (right) in wild-type mice was measured with prophylactic injection of GIP (374 μg/mL, blue) or saline (white). n = 7, 8 (left) and 7, 7 (right); mean ± SEM; circles: individual data points; Mann-Whitney test, **p < 0.01. (B and C) The area postrema of *Gipr-Cre; Rosa26-lsl-DTR* mice was injected with DT (+DT) or saline (−DT). Immunohistochemistry for DTR (B) and quantification of DTR-expressing cells (C) in coronal brain cryosections containing area postrema, paraventricular hypothalamus (PVH), and arcuate nucleus (ARC). Scale bar: 100 μm; n = 4–46 sections from 4 mice (−DT) or 12 mice (+DT); mean ± SEM; circles: individual data points; ****p < 0.0001. (D) GDF15-induced flavor avoidance was measured with prophylactic GIP injection (374 μg/mL) in non-ABLATE and *Gipr*-ABLATE^AP^ mice. n = 16, 12; mean ± SEM; circles: individual data points; *p < 0.05, Mann-Whitney test. (E) Cartoon depicting the mechanism of GIP action involving inhibitory GIPR neurons and excitatory GFRAL neurons in the area postrema.

GFRAL neurons mediate nausea-associated responses to several visceral threats (Zhang et al., 2021), so we reasoned that inhibiting GFRAL neurons through local area postrema circuits may provide an effective way to attenuate poison responses. Pharmacological access to cluster 6 neurons is facilitated as the area postrema contains a reduced blood-brain barrier and local neurons can directly detect circulating peptides like GIP. We tested whether GIP could suppress nausea-related behaviors and whether GIP-evoked behavioral responses required area postrema inhibitory circuits. Using the conditioned-flavor-avoidance paradigm described above, wild-type mice were administered either GIP or saline on the conditioning day and 20 min later were exposed to GDF15 or LiCl. GDF15 and LiCl evoked robust flavor-avoidance responses in saline-injected control mice, but responses were abolished in mice receiving a prophylactic GIP injection (Figure 4A).

We asked whether area postrema cluster 6 neurons were required for the suppression of nausea-related behavior by GIP. We used a genetic approach involving diphtheria toxin (DT) to ablate Cre-expressing GIPR neurons in the area postrema. Mouse cells are DT resistant but can be made susceptible by Cre-guided expression of the DT receptor (DTR) (Buch et al., 2005). DT was injected into the area postrema of *Gipr-Cre; Rosa26-lsl-DTR* mice (*Gipr*-ABLATE^AP^ mice) or of Cre-negative *Rosa26-lsl-DTR* mice (non-ABLATE mice) as a control. *Gipr*-ABLATE^AP^ mice displayed near-complete loss of cluster 6 neurons, but not other area postrema neuron types, and some loss of GIPR neurons in the NTS but not loss of GIPR neurons in other brain regions such as the hypothalamus (Figures 4B and 4C). Behavioral responses of *Gipr*-ABLATE^AP^ mice and non-ABLATE mice were examined using the conditioned-flavor-avoidance paradigm. We observed that GIP protected against GDF15-conditioned flavor avoidance in non-ABLATE mice but no longer suppressed GDF15 responses in *Gipr*-ABLA-TE^AP^ mice (Figure 4D). Thus, GIP suppresses nausea-associated behavior through area postrema inhibitory neurons.

## DISCUSSION

Nausea evoked by visceral poisons can be counterproductive, causing patients to forego life-saving medications for cancer, diabetes, and other diseases. Studies here establish area postrema inhibitory neurons (cluster 6, GIPR neurons) as a target for suppressing behavioral responses to at least some nausea-inducing toxins (Figure 4E). GIP is a gut hormone and incretin that activates GIPR neurons, and GIPR neurons in turn suppress the activity of nearby GFRAL-expressing and nausea-promoting excitatory neurons. In addition to GIPR, cluster 6 neurons express other cell surface receptors (Zhang et al., 2021), including mu-opioid receptor and neuropeptide Y receptor 2; it is possible that activating these receptors stimulates or inhibits GIPR neurons, with other stimulating agonists potentially providing additional avenues for pharmacological modulation of nausea-related behaviors with variable associated side effects. Furthermore, the strategy of targeting inhibitory circuits will likely be applicable to other area postrema neurons, including SLC6A2 neurons, which also condition flavor avoidance (Zhang et al., 2021) and receive distinct local inhibitory input from GAD2-positive, GIPR-negative neurons. Other inhibitory neurons (cluster 5) express the ghrelin receptor (Zhang et al., 2021), raising the possibility that several gut hormones act in concert to toggle the balance of area postrema inhibition and excitation. GIP is released following nutrient-rich meals (Baggio and Drucker, 2007), and it may be beneficial under certain physiological conditions for animals to consume calorie-rich foods containing small amounts of harmful chemicals. Area postrema circuits that integrate reward and punishment signals could guide future consummatory decisions based on need, reward value, and toxin risk. Together, experiments here reveal that GIP suppresses poison responses through a dedicated neuronal pathway, key insights into area postrema circuit organization, and a potential strategy for nausea intervention that involves targeting of brainstem inhibitory neurons.

### Limitations of the study

GIP directly stimulates cluster 6 neurons in slice preparations (Figure 2B), and peripheral GIP injection induces area postrema Fos expression (Figure S3B), but it has not been demonstrated that a sufficient concentration of meal-induced GIP will naturally arrive at the brainstem to stimulate area-postrema-localized GIPR. Area postrema GIPR neurons are required for GIP responses reported here (Figure 4D), yet a less parsimonious model is that GIP acts through an upstream neuronal route to engage area postrema GIPR neurons indirectly. GIP does not activate GIPR-negative area postrema neurons (Figures 2D and 3), and while the area postrema receives predominant input from the vagus nerve, area postrema GIP responses persist in vagotomized mice (Figure S3B). Additional studies are needed to understand when GIPR neurons are naturally engaged and whether they are activated by meal consumption, vagal inputs, top-down inputs, and/or other stimuli.

## STAR★METHODS

### RESOURCE AVAILABILITY

#### Lead contact

Further information and requests for resources and reagents should be directed and will be fulfilled by the Lead Contact, Stephen Liberles (Stephen_liberles@hms.harvard.edu).

#### Materials availability

All mouse lines are available as previously described (Zhang et al., 2021), except *Gipr-Cre* which is available from Frank Reimann upon reasonable request.

#### Data and code availability

- All primary images and data have been deposited at Mendeley Data (https://doi.org/10.17632/h3fztb3ynh.1).
- This paper does not report original code.
- Any additional information related to data reported is available from the lead contact upon request.

### EXPERIMENTAL MODEL AND SUBJECT DETAILS

All animal husbandry and procedures were performed in compliance with institutional animal care and use committee guidelines. All animal husbandry and procedures followed the ethical guidelines outlined in the NIH Guide for the Care and Use of Laboratory Animals (https://grants.nih.gov/grants/olaw/guide-for-the-care-and-use-of-laboratory-animals.pdf), and all protocols were approved by the institutional animal care and use committee (IACUC) at Harvard Medical School. *Slc6a2-p2a-Cre* (Zhang et al., 2021), *Gfral-p2a-Cre* (Zhang et al., 2021), *Glp1r-ires-Cre* (Chang et al., 2015; Williams et al., 2016), *Calcr-ires-Cre* (Pan et al., 2018), and *Gipr-Cre* (Adriaenssens et al., 2019) mice were described before; wild-type C57BL/6J (000664), *Rosa26-lsl-L10GFP* (024750), *Rosa26-lsl-tdTomato* (007908), *Gad2-ires-Cre* (010802), *Agtr1a-t2a-Cre* (031487), and *Rosa26-lsl-DTR* (007900) mice were purchased (Jackson Laboratory). Both male and female mice between 8–24 weeks old were used for all other studies, and no differences based on sex were observed.

### METHOD DETAILS

#### Electrophysiology and circuit mapping

Brain tissue was dissected from mice (6–10 weeks of age) following anesthesia (isoflurane) and decapitation, and brain slices (200 μm) were cut with a Leica VT1000S vibratome in slicing solution (4°C) [slicing solution: (in mM) 110 choline chloride, 2.5 KCl, 25 NaHCO_3_, 5 sodium ascorbate, 1.25 NaH_2_PO_4_, 7 MgCl_2_, 25 D-glucose, 0.5 CaCl_2_, 2 Ethyl pyruvate, continually bubbled with carbogen gas (95% O_2_/5%CO_2_)]. Then, slices were maintained and recorded at room temperature in oxygenated artificial cerebral spinal fluid (ACSF) solution [ACSF solution: (in mM) 126 NaCl, 3 KCl, 20 NaHCO_3_, 1.25 NaH_2_PO_4_, 2 MgCl_2_, 10 D-glucose, 2 CaCl_2_, continually bubbled with carbogen gas]. Inhibitory synaptic currents were recorded using a cesium methanesulfonate-based intracellular solution with high chloride, which contained (in mM): 120 CsCl, 15 CeMeSO_3_, 8 NaCl, 0.5 EGTA, 10 HEPES, pH 7.3, 290 mOsm. Photostimulation-evoked IPSCs were recorded in the whole-cell, voltage-clamp mode, with membrane potential clamped at −60 mV. To photo-stimulate channelrhodopsin-positive cells, an LED light source (473 nm; CoolLED, Andover, UK) was used and controlled by the pClamp 10.2 software (Axon Instruments), with a photostimulation involving five 50 ms blue light laser pulses administered 1 s apart. In some experiments, 10 μM bicuculline methiodide (Tocris 2503) and 100 nM tetrodotoxin (Tocris 1078) were applied during recordings. GIP (100 nM, [D-Ala^2^]-GIP, Tocris 6699) responses were measured by whole-cell recordings in current-clamp mode and zero holding current. Intracellular solution contained (in mM) 130 K-Gluconate, 15 KCl, 4 NaCl, 0.5 CaCl_2_, 10 HEPES, 1 EGTA, pH 7.2, 290 mOsm. As indicated, some experiments involved administration of cyanquixaline (CNQX, 10 μM, Tocris 1045), D-AP5 (50 μM, Tocris 0106), and bicuculline methiodide (10 μM). Recordings were obtained using borosilicate glass microelectrodes (2–5 MΩ) and Molecular Device 700B amplifier with data filtered at 1 kHz.

#### cAMP imaging of area postrema neurons

Neuronal responses were measured in acutely dissociated area postrema neurons. Brains were acutely harvested from *Gipr-Cre, Rosa26-lsl-tdTomato* mice. Coronal brain slices (200 μm, Leica VT1000S vibratome) containing the area postrema were obtained in slicing solution (4°C): (in mM) 110 choline chloride, 2.5 KCl, 25 NaHCO_3_, 5 sodium ascorbate, 1.25 NaH_2_PO_4_, 7 MgCl_2_, 25 D-glucose, 0.5 CaCl_2_, 2 Ethyl pyruvate, continually bubbled with carbogen gas (95%O_2_/5%CO_2_)]. The area postrema was visualized by microscopy and harvested based on anatomical landmarks. Harvested tissues were digested with a papain dissociation system (Worthington Biochemical, LK003150, 60 min, 37°C), washed [Earle’s Balanced Salt Solution containing ovomucoid protease inhibitor (1 mg/mL), BSA (1 mg/mL), and DNase (100 units/mL)], and gently triturated with three pipettes of decreasing diameters. Isolated single cells were centrifuged (100g, 5 min, 4°C) and resuspended in culture medium [Neurobasal medium with 2.5% fetal bovine serum, N-2 (Thermo Fisher 17502048), B-27 (Thermo Fisher 17504044), Glutamax (Thermo Fisher 35050061), and Antibiotic-Antimycotic (Thermo Fisher 15240062)]. Cells were plated on laminin-coated coverslips (neuVitro, GG-12-Laminin), and transfected (overnight, 37°C) with a viral vector encoding Green Up cADDis cAMP sensor (Montana Molecular U0200G). Real-time cAMP transients were imaged using a Leica SP5 II confocal microscope with cells under continuous gravity-based perfusion (0.6 mL/min flow rate, 1 mL chamber reservoir) of Ringer’s solution (in mM 140 NaCl, 5 KCl, 2 MgCl_2_, 2 CaCl_2_, 10 HEPES, 10 glucose, pH 7.4) alone or containing 100 nM [D-Ala^2^]-GIP or 25 μM forskolin (Tocris 1099). Responses were processed with Matlab (MathWorks), and for display, smoothened with the smoothdata function, as a moving average with an automated window length. The baseline activity for each neuron was defined as the average green fluorescence intensity over a five-minute period preceding stimulus delivery, and cells were excluded if they failed to display responses to positive controls.

#### AAV/DT injections

Mice were anaesthetized (avertin) and placed on a stereotaxic frame (David Kopf Instruments) with heads facing down ~45°. The fourth ventricle and area postrema were surgically exposed after removal of the meninges, and a Nanoject III Injector (Drummond) was positioned directly into the area postrema for injections of virus (titer ≥ 10^13^ vg/mL, 35 nL, 2 nL/second) or DT (Sigma D0564, 5 μg/mL in saline, 35 nL, 2 nL/second). Animals recovered for 3–4 weeks for behavioral analysis. Injected viruses included AAV-Flex-ChR2-mCherry (Addgene, AAV9-EF1A-DIO-hChR2(H134R)-mCherry, prep #20297-AAV9), AAV-Flex-Gα_s_-DREADD (Addgene, AAV-SYN-DIO-rM3D (Gα_s_)-mCherry, plasmid 50458, custom virus (Zhang et al., 2021), serotype 9), AAV-Flex-GFP (Addgene, AAV9-pCAG-Flex-EGFP (Oh et al., 2014), prep #51502-AAV9), AAV-Flex-synaptophysin-mCherry (MGH GDT core, AAV-RN1: AAV8.2-EF1a-DIO-synaptophysion-mCherry). After behavioral measurements, tissue was collected for analysis of 1) ablation efficiency by DTR immunohistochemistry or 2) viral reporter expression. Animals with incomplete DT-induced ablation or that lacked AAV-mediated reporter expression in the area postrema were determined by an investigator blind to behavioral results, and were excluded from subsequent analysis; the numbers of excluded animals were (left to right) 0, 3, 0, 3, 0, 9 in Figure 1E and 0, 17 in Figure 4D.

#### Analysis of RNA, protein, and reporter expression

Hybridization chain reaction (HCR) RNA *in situ* hybridization was performed on cryosections of unfixed brain (25 μm) following the HCR-FISH 3.0 protocol (Choi et al., 2018, 2020). *Gipr* probe, probe hybridization buffer, probe wash buffer, amplification buffer, and fluorescent HCR hairpins were purchased from Molecular Instruments (Los Angeles, USA). *Gipr* probe was designed to be associated with the B3 initiator sequence and detected by hairpins labeled with Alexa Fluor 647 (Lot# PRL060). Fluorescent images were analyzed with a Leica SP5 II confocal microscope.

Immunohistochemistry and reporter fluorescence analysis was performed on cryosections (40 μm) of tissues fixed by intracardial perfusion with cold fixative (4% paraformaldehyde, PBS) and cryopreserved (30% sucrose, PBS, 4°C, two days). Slide-mounted sections were blocked (1 hr, RT, blocking solution: PBS, 5% donkey serum, 0.1% Triton X-100), incubated with primary antibody (blocking solution, 4°C, overnight), washed (300:1, PBST, 3 × 5 min, RT), incubated with fluorophore-conjugated secondary antibodies (PBST, 5% donkey serum, 1–2 hours, RT). Antibody solutions were Rabbit-anti-RFP (for mCherry, Rockland AB_2209751, 1:1000), Chick-anti-GFP (for GFP, Aves Labs GFP-1020, 1:1000), Goat-anti-HB-EGF (for DTR, R&D Systems AF-259-NA, 1:500). Fluorescent images were analyzed with a Leica SP5 II confocal microscope and processed with FIJI (Schindelin et al., 2012).

#### Behavioral assays

Conditioned flavor avoidance assays involved a seven-day protocol with daily 30-minute introductions to a test arena containing two water bottles soon after dark onset. Mice had no access to water in the home arena but given *ad libitum* water access in the test arena. In the first three days (habituation days), both water bottles contained unflavored water. On day four (conditioning day), both water bottles were filled with either grape-flavored or cherry-flavored water (grape or cherry Kool-Aid) sweetened with 0.2% saccharin, each on half of the trials. Immediately after test arena occupancy on the conditioning day, mice were injected with saline (10 μL/g, IP) alone or containing either CNO (1 mg/kg, Tocris 6329), GDF15 (20 μg/kg, R&D systems 957-GD-025/CF), [D-Ala^2^]-GIP (374 μg/kg), or LiCl (168 mg/kg). On day five (recovery day), both water bottles contained unflavored water. On day six (testing day 1), one water bottle contained cherry-flavored water, while the other contained grape-flavored water on a randomized arena side, and consumption from each water bottle scored using an automated lickometer based on a published design (Slotnick, 2009). On day seven (testing day 2), the positions of the two bottles containing either grape- or cherry-flavored water were switched. Preference index was calculated as time drinking the conditioned flavor water divided by total time drinking and was based on the first test day results.

Fos studies involved IP injection of CNO (1 mg/kg, Tocris 6329) or [D-Ala^2^]-GIP (374 μg/kg) 2.5 hours before tissue harvest. For Figure S3B, bilateral vagotomy was performed to remove the right cervical trunk and left subdiaphragmatic vagal trunk; seven days later, Fluoro-Gold (Santa Cruz sc-358883, 1% in saline, 5 μL/g) was injected IP and after another three days, GIP-induced Fos was measured.

### QUANTIFICATION AND STATISTICAL ANALYSIS

All data points are derived from different mice except for some data points related to expression quantification (Figures 4C and S2B) which include multiple sections per mouse, and recordings (Figures 2D, 3C and S1A) which include multiple cells per mouse. Sample sizes from left to right: Figure 1C (5 cells from 5 mice), Figure 1D (18 cells from 5 mice), Figure 1E (5, 6, 9, 12, 9, 12), Figure 2D (17, 74), Figure 3C (14, 7, 15, 15, 12, 10, 13, 11, 20, 15), Figure 4A (7, 8, 7, 7), Figure 4C (6, 46, 7, 46, 4, 11, 4, 9), Figure 4D (16, 12), Figure S1A (32), Figure S1B (16), Figure S1C (22 cells from 5 mice), Figure S1D (21 cells from 5 mice), Figure 2D (15, 15, 15, 15). All graphs report mean ± SEM as indicated in figure legends.

In neuronal imaging experiments, cells were counted as responsive if stimulus-evoked Δ*F/F* exceeded three standard deviations above the baseline mean. Statistical significance was measured on Prism 9 software (Graphpad) using a Mann-Whitney test (Figures 1E, 4A and 4D) and a Mann-Whitney test with Bonferroni correction (Figure 4C).

## Supplementary Material

1

## Figures and Tables

**Table:** KEY RESOURCES TABLE

REAGENT or RESOURCE	SOURCE	IDENTIFIER
Antibodies		
Rabbit-anti-RFP	Rockland	Cat# 600-401-379; RRID:AB_2209751
Goat-anti-HB-EGF (anti-DTR)	R&D Systems	Cat# AF-259-NA, RRID:AB_354429
Rabbit-anti-Fos	Synaptic Systems	Cat# 226 003, RRID:AB_2231974
Chick-anti-GFP	Aves Labs	Cat# GFP-1020, RRID:AB_10000240
Bacterial and virus strains		
AAV9-pCAG-Flex-EGFP	Oh et al., 2014	Addgene, Cat #51502-AAV9
AAV9-Ef1a-DIO-hChR2(H134R)-mCherry	Gift from Karl Deisseroth	Addgene, Cat #20297-AAV9
pAAV-hSyn-DIO-rM3D (Gα_s_)-mCherry	Gift from Bryan Roth	Addgene, Cat #50458
AAV8.2-hEF1a-DIO-synaptophysin-mCherry	MGH Viral Core	Cat #AAV-RN1
Green Up cADDis cAMP sensor	Montana Molecular	Cat #U0200G
Chemicals, peptides, and recombinant proteins		
[D-Ala^2^]-GIP	Tocris	Cat #6699
Clozapine N-oxide dihydrochloride	Tocris	Cat #6329
Bicuculline methiodide	Tocris	Cat #2503
Cyanquixaline	Tocris	Cat #1045
Forskolin	Tocris	Cat #1099
Diphtheria Toxin from *Corynebacterium diphtheriae*	Sigma-Aldrich	Cat #D0564
D-AP5	Tocris	Cat #0106
GDF15	R&D systems	Cat #957-GD-025/CF
Tetrodotoxin	Tocris	Cat #1078
*Gipr* B3 probe	Molecular Instruments	Lot #PRL060
FluoroGold	Santa Cruz Biotechnology	Cat #sc-358883
Critical commercial assays		
Papain dissociation system	Worthington Biochemical	Cat # LK003150
Deposited data		
Raw images	This study	Mendeley data https://doi.org/10.17632/h3fztb3ynh.1
Experimental models: Organisms/strains		
*Glplr-ires-Cre*	Chang et al., 2015; Williams et al.,2016	Deposited in Jackson laboratory (Cat#029282)
*Calcr-ires-Cre*	Pan et al., 2018	N/A
*Gipr-Cre*	Adriaenssens et al., 2019	N/A
Wild-type C57BL/6J	Jackson Laboratory	Cat #000664
*Rosa26-LSL-L10GFP*	Jackson Laboratory	Cat #024750
*Agtr1a-t2a-Cre*	Jackson Laboratory	Cat #031487
*Rosa26-LSL-tdTomato*	Jackson Laboratory	Cat #007908
*Gad2-ires-Cre*	Jackson Laboratory	Cat #010802
*Rosa26-LSL-DTR*	Jackson Laboratory	Cat #007900
*Slc6a2-p2a-Cre*	Zhang et al., 2021	N/A
*Gfral-p2a-Cre*	Zhang et al., 2021	N/A
Software and algorithms		
MATLAB R2019a	MathWorks	RRID: SCR_001622 https://www.mathworks.com/products/matlab.html
PRISM 9	GraphPad	RRID: SCR_002798 https://www.graphpad.com/scientific-software/prism/
FIJI	Schindelin et al., 2012	RRID:SCR_002285 https://fiji.sc
pClamp 10.2	Axon Instruments	RRID: SCR_011323 https://www.moleculardevices.com/products/software/pclamp.html
BioRender	BioRender.com	RRID: SCR_018361 https://biorender.com

## Highlights

- A transcriptome-defined area postrema inhibitory neuron type expresses GIPR
- GIPR neurons project locally and selectively inhibit nausea-related GFRAL neurons
- GIP activates inhibitory neurons, blocks GFRAL neurons, and suppresses nausea
- Ablating brainstem GIPR neurons removes anti-nausea effects of GIP
