# Supplementary material for: A brainstem circuit for nausea suppression
Source: Cell Rep. Author manuscript; Available in PMC 2022 Jul 7. (PMC9260880; doi:10.1016/j.celrep.2022.110953)
Supplement: 1 [file NIHMS1816590-supplement-1.pdf]

Cell Reports, Volume *39*

Supplemental information

A brainstem circuit for nausea suppression

Chuchu Zhang, Lindsay K. Vincelette, Frank Reimann, and Stephen D. Liberles

Supplementary Figure 1

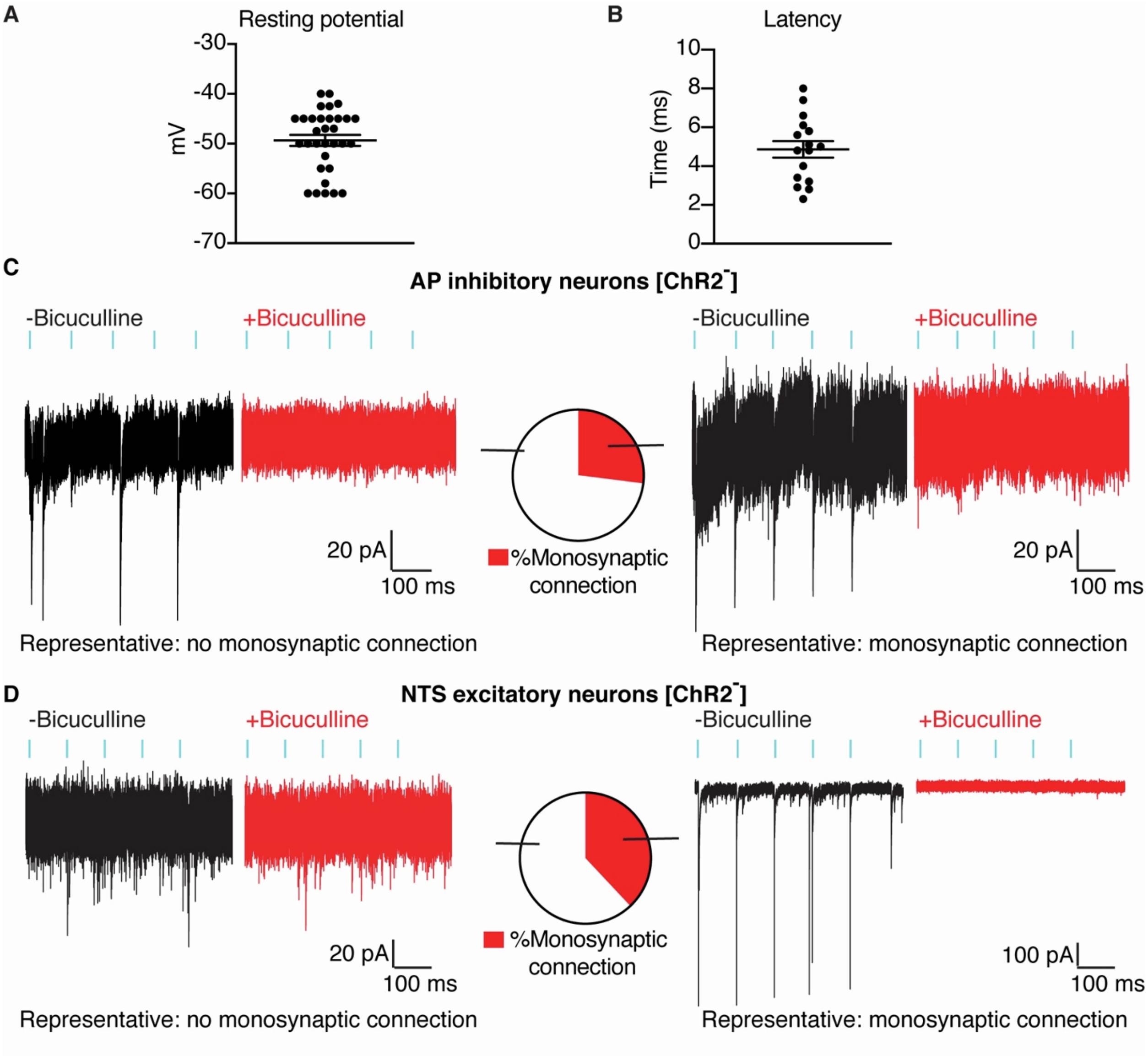

Figure S1. Connectivity patterns of area postrema inhibitory neurons, related to Figure 1.
(A) Resting membrane potential of area postrema excitatory neurons recorded using whole-cell, patch-clamp with a potassium gluconate-based intracellular solution, n=32, mean ± sem, circles: individual data points.
(B) Latency of postsynaptic currents in area postrema excitatory neurons after onset of light stimulation to activate area postrema inhibitory neurons, from data in Figure 1D, n=16, mean ± sem, circles: individual data points.
(C) Light-induced postsynaptic currents were measured in GFP-positive mCherry-negative area postrema neurons from *Gad2-ires-Cre, Rosa26-lsl-L10GFP* mice previously injected in the area postrema with AAV-Flex-ChR2-mCherry. Whole-cell, voltage-clamp recordings of photostimulation-induced IPSCs were made at -60 mV using a high chloride intracellular solution to reveal a chloride conductance. Recordings were made with (red) or without (black) bicuculline (10 µM). Representative traces indicating the presence (right) or absence (left) of monosynaptic connections and quantification (middle, monosynaptic connections observed in 6/22 neurons from 5 mice).
(D) Light-induced postsynaptic currents were measured in GFP-negative NTS neurons from *Gad2-ires-Cre, Rosa26-lsl-L10GFP* mice previously injected in the area postrema with AAV-Flex-ChR2-mCherry. Recordings were made with (red) or without (black) bicuculline (10 µM). Whole-cell, voltage-clamp recordings of

photostimulation-induced IPSCs were made at -60 mV using a high chloride intracellular solution to reveal a chloride conductance. Representative traces indicating the presence (right) or absence (left) of monosynaptic connections and quantification (middle, monosynaptic connections observed in 8/21 neurons from 5 mice).

Supplementary Figure

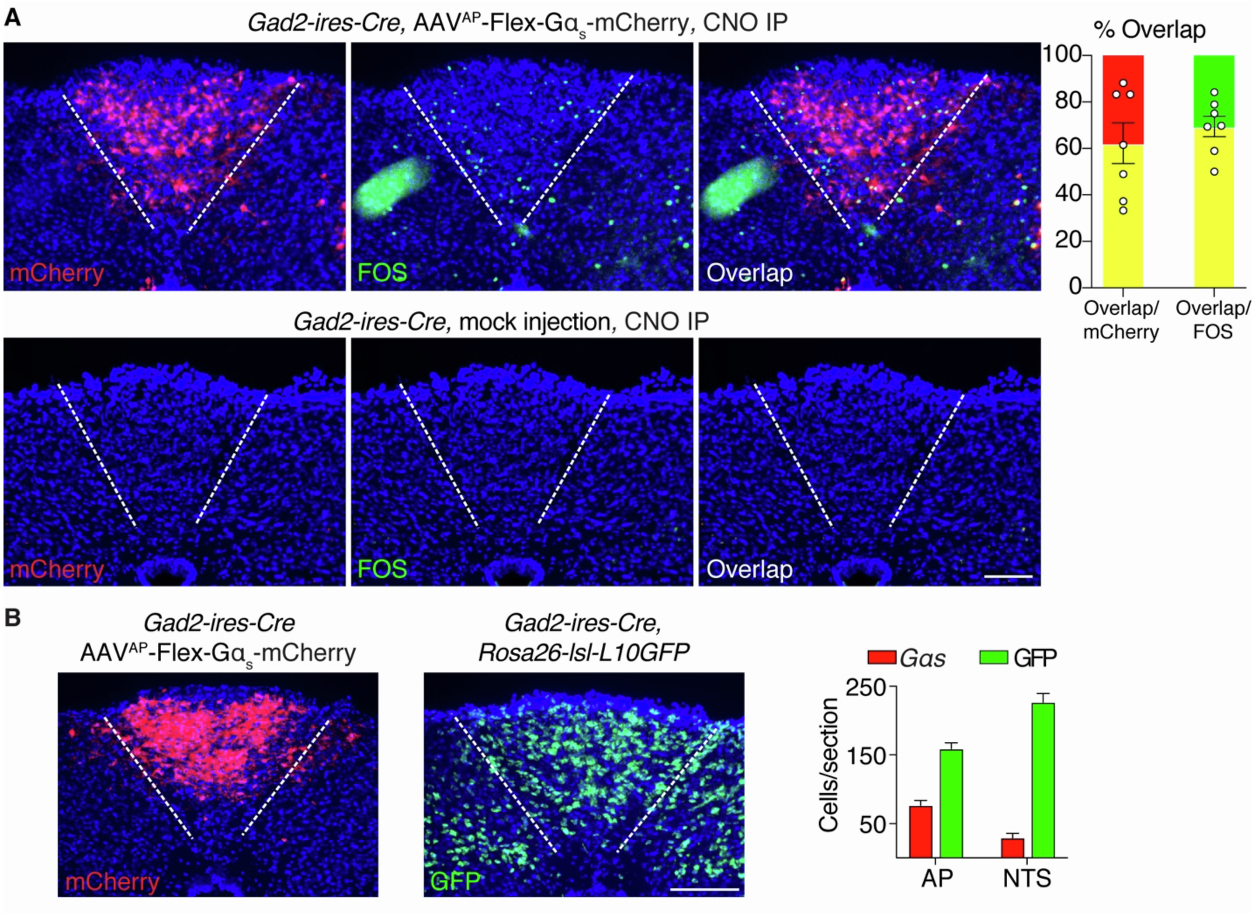

Figure S2. Validation of chemogenetic approaches to activate area postrema inhibitory neurons, related to Figure 1.

(A) *Gad2-ires-Cre* mice were injected in the area postrema with (top) or without (bottom) AAV-Flex-GsDREADD-mCherry. CNO was injected IP, and two-color expression analysis was subsequently performed in fixed coronal area postrema cryosections for native mCherry fluorescence (red) and cFos immunofluorescence (green), scale bar: 100 µm. Fos responses were observed in mCherry-labeled area postrema neurons of mice injected with AAV-Flex-GsDREADD-mCherry (quantification on the right), but were not observed in control mice lacking AAV-Flex-GsDREADD-mCherry.

(B) *Gad2-ires-Cre* mice were either injected in the area postrema with AAV-Flex-GsDREADD-mCherry (left) or crossed to mice with a *Rosa26-lsl-L10GFP* allele (middle), scale bar: 100 µm. Quantification (right) of area postrema (AP) or NTS cells displaying native reporter fluorescence per coronal brainstem cryosection (40 µm) from *Gad2-ires-Cre* mice injected in the AP with AAV-Flex-GsDREADD-mCherry mice (red) or *Gad2-ires-Cre*; *Rosa26-lsl-L10GFP* mice (green), n=15 sections from 10 ($G\alpha_s$) or 7 (GFP) mice.

Supplementary Figure 3

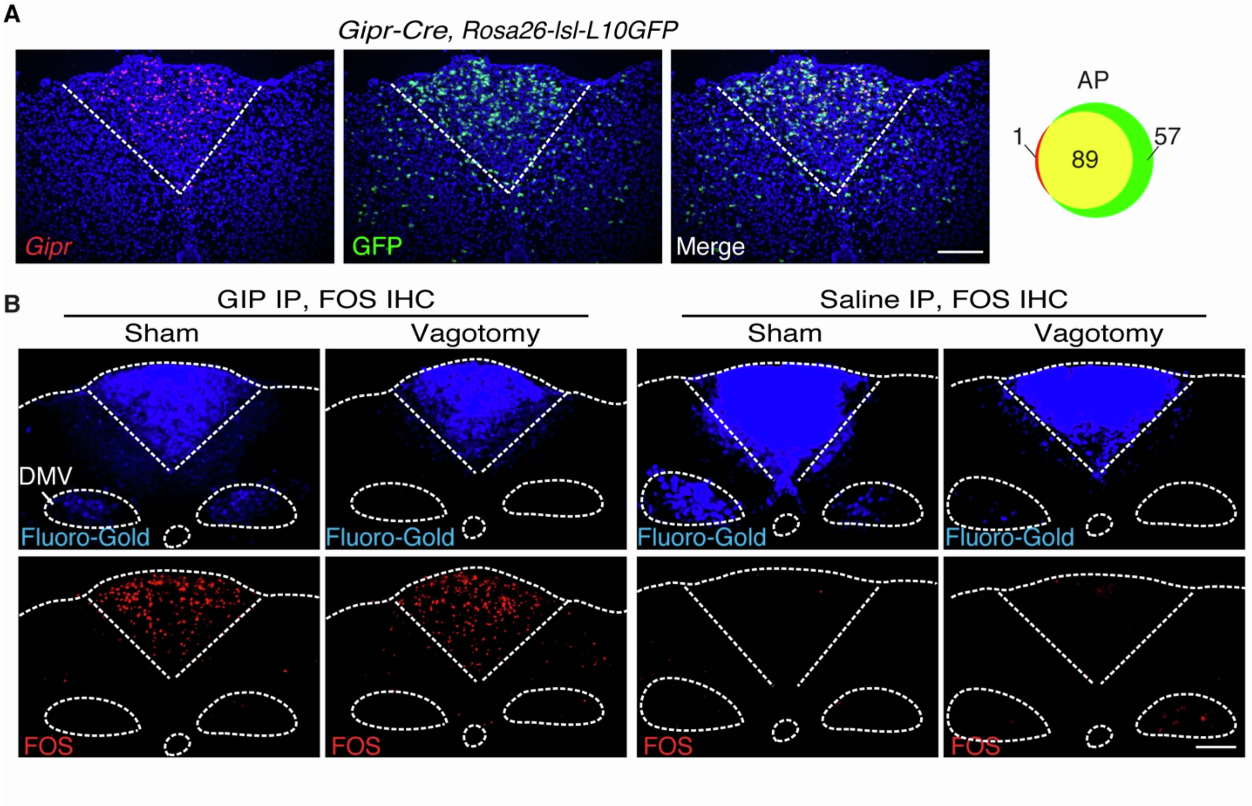

Figure S3. Validation of *Gipr-Cre* mice, related to Figure 2.
(A) Two-color expression analysis in coronal area postrema cryosections of *Gipr-Cre, Rosa26-lsl-L10GFP* mice, with RNA *in situ* hybridization to detect *Gipr* transcripts (red) and native GFP fluorescence (green), scale bar: 100 µm. The numbers of co-labeled (yellow) or individually labeled (red, green) cells were counted (right).
(B) Mice underwent either sham surgery or bilateral vagotomy (unilateral cervical vagotomy and unilateral subdiaphragmatic vagotomy), and were injected (IP) with Fluoro-Gold to label uncut neurons in the dorsal motor nucleus of the vagus (DMV). Mice were later injected (IP) with either GIP or saline, and fixed coronal brainstem sections were harvested for Fos immunochemistry and visualization of native Fluoro-Gold fluorescence, scale bar: 100 µm.
